# Validity of Thermal Ramping Assays Used to Assess Thermal Tolerance in Arthropods

**DOI:** 10.1371/journal.pone.0032758

**Published:** 2012-03-09

**Authors:** Johannes Overgaard, Torsten Nygaard Kristensen, Jesper Givskov Sørensen

**Affiliations:** 1 Department of Bioscience, Aarhus University, Aarhus, Denmark; 2 Department of Molecular Biology and Genetics, Aarhus University, Tjele, Denmark; University of Arizona, United States of America

## Abstract

Proper assessment of environmental resistance of animals is critical for the ability of researchers to understand how variation in environmental conditions influence population and species abundance. This is also the case for studies of upper thermal limits in insects, where researchers studying animals under laboratory conditions must select appropriate methodology on which conclusions can be drawn. Ideally these methods should precisely estimate the trait of interest and also be biological meaningful. In an attempt to develop such tests it has been proposed that thermal ramping assays are useful assays for small insects because they incorporate an ecologically relevant gradual temperature change. However, recent model-based papers have suggested that estimates of thermal resistance may be strongly confounded by simultaneous starvation and dehydration stress. In the present study we empirically test these model predictions using two sets of independent experiments. We clearly demonstrate that results from ramping assays of small insects (*Drosophila melanogaster*) are not compromised by starvation- or dehydration-stress. Firstly we show that the mild disturbance of water and energy balance of *D. melanogaster* experienced during the ramping tests does not confound heat tolerance estimates. Secondly we show that flies pre-exposed to starvation and dehydration have “normal” heat tolerance and that resistance to heat stress is independent of the energetic and water status of the flies. On the basis of our results we discuss the assumptions used in recent model papers and present arguments as to why the ramping assay is both a valid and ecologically relevant way to measure thermal resistance in insects.

## Introduction

Researchers investigating thermal tolerance in ectothermic animals are often challenged when designing appropriate laboratory assays. This is partly due to an inherent time by temperature interaction where thermal stress accumulates over time but also because particular time-temperature combinations can elicit or fail to elicit plastic responses. Time therefore impacts on thermal tolerance measures because loss of function or mortality may be induced by either short and extreme temperatures or through longer and more moderate temperature exposures [1]–[6]. Given this interaction it becomes difficult to define a universal and “true” thermal limit. This problem is further accentuated by the fact that animals display marked hardening and acclimation responses where even very short pre-exposures to heat or cold may alter the inherent thermal tolerance [7]–[9]. Instead of addressing these issues directly by measuring a matrix of thermal resistances using different exposure times and temperatures, several research groups have chosen to integrate these “problems” into their tests by exposing test animals to temperatures that are gradually increased or decreased – so called ramping assays [4], [10]–[14]. The most important argument for using ramping assays is that gradual exposures are more “ecologically relevant”, i.e. animals are exposed to gradually changing temperatures in their natural environment (recently reviewed by [15]). It has been highlighted how gradual exposure to increasing or decreasing temperatures allows time for the expression of physiological coping mechanisms [4], [14] and depends on the actual rate of temperature change [11], [12], [16]. This might not be possible in assays where animals are exposed abruptly to extreme temperatures [15].

**Figure 1:**
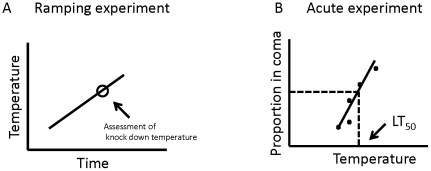
Schematic representation of thermal tolerance assays. A) Ramping procedure where temperature is gradually increased and the critical thermal limit (CTmax) is registered as the temperature at which the animal is knocked out. In this study we employed two ramping rates of 0.1 and 0.06°C min^−1^, respectively. B) Acute thermal tolerance is inferred from the temperature giving 50% acute knock down (flies are knocked down within 5 min). By exposing groups of flies to a range of temperatures close to that giving 50% knock down, the acute CTmax is estimated from linear regression.

The ramping approach was recently criticized by Rezende et al. [17] and Santos et al. [18] who proposed that the estimate of thermotolerance obtained from ramping techniques is confounded by simultaneous and unwanted exposure to starvation and dehydration stress. Rezende et al. [17] and Santos et al. [18] especially emphasized that slow ramping of temperature is problematic since this will inherently increase exposure time. This issue arises because ramping assays commonly are performed by placing individual insects in small closed containers that are submerged in a water bath at a benign temperature after which the temperature is gradually raised/decreased (Fig. 1). These containers do typically not contain food or water. The models presented by Rezende et al. [17] and Santos et al. [18] therefore predict that water loss and energy expenditure impact (underestimate) the “true” thermal tolerance when this is obtained from a ramping assay. They conclude that the problems associated with starvation and desiccation are so profound that the ramping method should generally be avoided (or at least the rate of temperature change should be high). However these conclusions were subsequently challenged in a comprehensive review by Terblanche et al. [15] which summarized the existing literature regarding thermal tests of ectothermic animals and tested some of the assumptions of the models used by Rezende et al. [17]. The present paper adds to this discussion by specifically testing the predictions formulated by Rezende et al. [17]. Thus in addition to focusing on *D. melanogaster*, the focal species of the Rezende et al. [17] and Santos et al. [18] papers, we also directly investigates the putative impact of starvation and dehydration by measuring water and energy stores of the insects before and after thermal tests and we discuss some of the assumptions presented in the previous model papers.

Based on empirical evidence we challenge the conclusions drawn by Rezende et al. [17] and Santos et al. [18] in two ways. First we use empirical data to investigate how ramping rate, starvation and dehydration affect the estimate of heat tolerance in *D. melanogaster*. These tests are designed to *i*) investigate if access to water and food during gradual heat exposure alleviates the heat stress experienced during ramping experiments and *ii*) investigate if acute measures of heat tolerance are affected by pre-exposure to starvation and dehydration. Second we discuss the assumptions used by Rezende et al. [17] and Santos et al. [18] and propose that different conclusions would have been reached in these studies if more realistic assumptions were used in their models. Based on these findings and arguments we conclude that ramping assays are both valid and ecologically relevant measures to assess thermal tolerance in *D. melanogaster* and similar sized arthropods. Although low environmental humidity is an important stressor for small insects and although high temperature severely limits desiccation tolerance our results show that moderate desiccation and starvation does not limit heat tolerance *per se* during short term stress exposures experienced during ramping assays. We believe this finding is relevant for the interpretation of how multiple environmental stressors affect the fundamental niche of insects.

## Materials and Methods

### Experimental protocol for the empirical experiments

**Figure 2:**
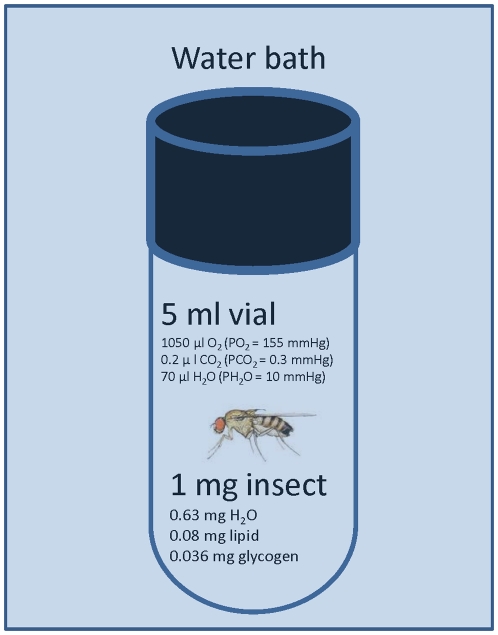
An example of the experimental conditions typically used during a ramping experiment. A small insect (here exemplified by a *D. melanogaster* female with a body-mass of 1 mg) is placed in a small closed container (5 ml). The amount of oxygen, carbon dioxide and water vapour are estimated using the assumption that the fly is loaded into the container under conditions of normal barometric pressure (760 mmHg), a room temperature of 20°C and a relative humidity of 50%. The total water, lipid and glycogen content are calculated on the basis of the empirical observations in this paper. As highlighted by recent studies [15], [17], [18] it is important to consider how thermal testing can potentially affect the energy and water resources of animals and this is largely determined by the fluxes of water and energy in the animal and obviously also by the conditions under which the animals are tested. Immediately after the container is closed the internal conditions in the container will start to change as a consequence of the animal's respiration and transpiration and as a consequence of the change in temperature. Thus, the animal will produce CO_2_ and water and use O_2_ while catabolizing its metabolic energy reserves. The animal will also exchange water over the respiratory and cuticular surfaces in a manner that is proportional to the product of whole animal “conductance” and the driving force for this water loss (the difference in partial pressure of water between the animal and its surroundings).

To test for the potential confounding role of food and water deprivation during assessment of maximal thermal tolerance (CTmax) in a ramping assay we performed two sets of experiments. The focus on CTmax (and not critical thermal minimum temperature) was chosen as high temperatures accelerate metabolic rate and water loss and therefore CTmax is more likely to be influenced by these confounding effects (See Fig. 2 for an presentation of the major changes expected under these experimental conditions). In the first experiment we exposed *D. melanogaster* to a ramping experiment and compared the CTmax as well as the level of dehydration and energy depletion in flies with and without access to food and water during the test. In the second set of experiments we pre-exposed flies to dehydration and/or starvation stress prior to a test of the acute CTmax. This was done to examine if/how water and energy status of the animal affected acute heat tolerance.

A mass bred laboratory population of *D. melanogaster* was used in the experiment. This population was created by mixing flies from 150 isofemale lines. The isofemale lines were started in May 2009 using F1 of flies collected in Coffs Harbour (New South Wales, Australia) and were maintained for five generations before the mass bred population was created. In all generations they were reared on a standard oatmeal-sugar-yeast-agar *Drosophila* medium under low to moderately high larval density conditions at 25°C, relative humidity (RH) of 50% and 12 h light/12 h dark cycles. Flies used in the experiments also developed at 25°C.

Experimental animals were controlled for density during development by transferring 50 eggs into each vial (7 ml medium) resulting in a density of 45 to 50 larvae per vial. Two to three days after eclosion the flies were briefly anesthetised under CO_2_ and mated females were collected for the experiments. The female flies were then left to recover for 2–3 days before the experiments were performed.

### Measuring CTmax using temperature ramping with and without access to food and water

It must be assumed that duration and therefore reduced ramping rate will exacerbate the potential problems associated with starvation and dehydration [15], [17], [18]. Thus, to test for the effects on CTmax of food and/or water deprivation during such test we performed two separate ramping assays where the flies were exposed to gradually increasing temperature at a rate of 0.06 and 0.1°C min^−1^, respectively. In each experiment a total of 75 individual female flies were quickly transferred to small screw-top glass vials (5 ml) without the use of anaesthetics. Flies were randomly allocated to one of three treatment groups (N = 25 flies) where controls were placed in empty vials, while the other two groups had access to either a droplet of agar (providing water) or fly food (providing water and food) that was placed in the lid. Vials were marked, and randomly placed in a rack which then was submerged in a temperature controlled water tank at 25°C. The water in the tank was continuously stirred by a pump to ensure homogeneity of the water temperature in the tank and the gradual change of temperature was initiated immediately after vials were submerged in the water bath. Flies were continuously monitored to register the temperature at which they were totally immobilized (knock down temperature - CTmax) (Fig. 1A).

### Measuring acute CTmax after pre-exposure to starvation and dehydration

To test the effects of starvation and dehydration on acute temperature tolerance we tested starved, dehydrated and control flies using a 5 min acute test. Prior to experiments the flies had been divided randomly into 7 experimental groups. One group remained under control conditions in food vials. Three groups received 3, 6 or 9 h of desiccation, respectively. Here flies were placed in empty vials in a large container with silica gel desiccant (RH<5%) at a constant temperature of 25°C. The last three experimental groups were placed in vials containing a water and agar mixture whereby they had access to water, but no access to food. Flies from these groups were exposed to starvation for 4, 10 and 16 h before the experiments were performed, respectively.

To measure the acute upper thermal limit, we placed individual flies in 5 mL glass vials and transferred groups of 5 flies directly to a water bath preset to a temperature aimed at giving more, less or approximately 50% knock down after 5 min of exposure. The proportion of flies knocked down was scored and the procedure was repeated (5–7 times) over a range of temperatures for each treatment group to allow for an assessment of the temperature at which 50% of the flies were knocked down as estimated from linear regression of the data (Fig. 1B).

### Lipid and water content

To determine the effects of temperature ramping on the water and energy budget of the flies a similar set of ramping assays (using the same procedure as described above) were run subsequently. Here we tested 50 female flies in each of the 3 experimental groups (agar (water), food and empty vials). Flies were exposed to gradually increasing temperatures until CTmax was reached where-after they were flash frozen in liquid N_2_ before they were weighed to nearest µg using a micro balance. From each experimental group we obtained 6 samples containing 7–11 flies. The samples were subsequently dried for 24 h at 60°C for determination of dry mass. Subsequently the dried flies were washed repeatedly in petroleum ether, and then re-dried to obtain lean mass and total lipid content. Finally, the glycogen content was measured as described below. Measurements of water, lipid and glycogen content for the 7 experimental groups in the acute experiment were performed in a similar manner.

### Glycogen measurements

Glycogen content was measured as described by Overgaard et al. [19]. In brief, 0.4 mL of 1 M NaOH was added to the sample vial and the sample was mechanically homogenised. Hereafter the samples were heated to 80°C for 3 h, thus extracting glycogen while degrading free glucose. Glycogen in the sample was subsequently digested to glucose for 2 h at 25°C in acetate buffer (0.25 M, pH 4.5) containing 400 mg L^−1^ amyloglucosidase (EC 3.2.1.3). Quantification of the glycogen store was carried out using a spectrophotometrically based enzymatic test kit for glucose (Glucose Gluc-DH, Diagnostic systems GmbH, Holzheim, Germany) and calculated relative to glycogen standards that had been subjected to the same extraction procedure as tissue samples.

### Statistics

Differences in thermotolerance, dry mass and water-, lipid- and glycogen- content between experimental groups in the ramping experiment were tested with a two-way ANOVA using the factors rate (0.06 or 0.1°C min^−1^) and treatment (empty vials, agar (water) or food). Differences in dry mass and water-, lipid-, and glycogen content between experimental groups used to assess acute CTmax were tested using a one-way ANOVA. Data of acute heat tolerance were fitted to a generalized linear model (glm) assuming a binomial distribution and using a logit-link function (logistic regression) using the open source statistical software package R v. 2.13.1 (http://www.r-project.org). Test for significance of temperature and treatment was performed using Wald ‘chi square’ tests. Associated LT_50_ (with S.E.M) were calculated for each treatment by the dose.p function in R. Unless otherwise mentioned all values are mean ± S.E.M. and statistical differences are inferred from P<0.05.

## Results

### Measuring CTmax using temperature ramping with and without access to food and water

**Figure 3:**
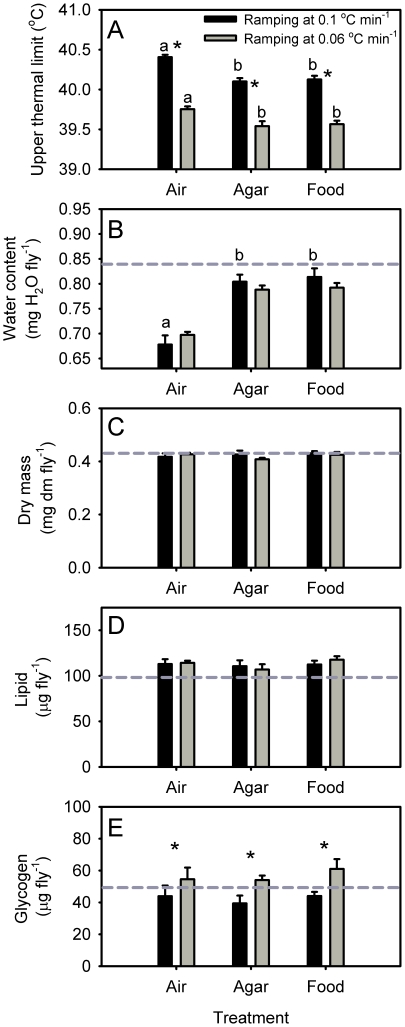
Temperature of heat knock down (A), water content relative to dry mass (dm) after thermal test (B), dry mass (dm) (C), percentage lipid (D) and glycogen content (E) in flies (*D. melanogaster*) exposed to gradual heating from 25°C using a rate of 0.1°C min^−1^ (black) and 0.06°C min^−1^ (grey), respectively. Flies were divided in three experimental groups with access to food, water (agar) or placed in empty vials (air). Statistical significant differences in relation to ramping rate are indicated with an asterisk and differences between treatment groups (food, agar or air) are illustrated by dissimilar letters.

CTmax is highly dependent on the methodology used. As seen in Fig. 3A CTmax was significantly higher (P<0.001) in flies exposed to the fast (0.1°C min^−1^) compared to the slower (0.06°C min^−1^) ramping regime. Interestingly, we found that CTmax was significantly higher (P<0.001) for flies placed in empty vials compared to flies with access to either food or water (agar). This difference was found under both ramping regimes such that the CTmax was 0.2–0.3°C higher for the flies tested in empty vials. Flies exposed to heat stress in empty vials also lost significantly more water (Fig. 3B, P<0.001) than flies with access to food or water. We found no treatment differences between our 3 experimental groups with regard to dry mass, lipid or glycogen content after the heat exposure (Figs. 3C–E). However, flies subjected to fast ramping (0.1°C min^−1^) had a glycogen content that was significantly lower than flies ramped at a slower rate (0.06°C min^−1^).

### Measuring acute CTmax after pre-exposure to starvation and dehydration

**Figure 4:**
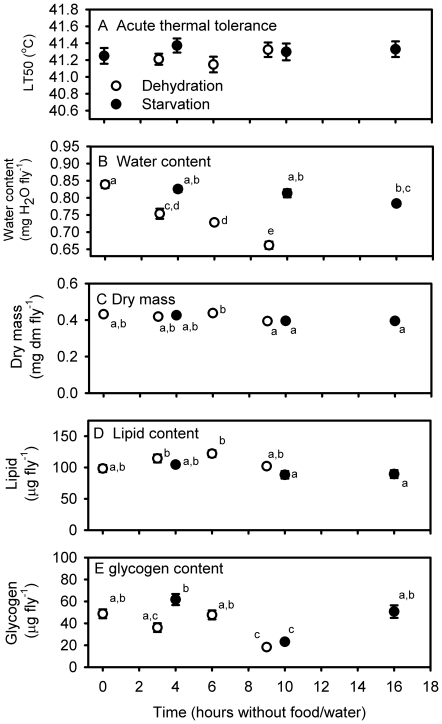
Temperature of acute heat knock down (A), water content relative to dry mass (dm) prior to thermal test (B), dry mass (dm) (C) and percentage lipid (D) and glycogen content (E) in flies (*D. melanogaster*) exposed acutely to high temperature. Statistical significant differences between treatment groups are indicated with dissimilar letters.

The proportion of flies knocked down by acute heat exposure was highly dependent on temperature (P<0.0001) for all treatment groups. However our estimates of CTmax (from the temperature at which 50% of the flies are knocked down) were independent of pre-treatment prior to the test (Fig. 4A). Thus all estimates fell close to 41.2–41.3°C and there were no significant differences between treatment groups. The pre-treatments significantly affected the dry mass, water-, lipid- and glycogen content of flies (Figs. 4B–E). Water content fell significantly after 3 h of dehydration and was lowest after 6 and 9 h of dehydration, while the flies with access to agar (i.e. starved but not dehydrated) did not differ from flies held at control conditions with access to food. There was a tendency for dry mass to decrease with exposure to starvation/dehydration and this was also partially manifested in a reduced lipid content after prolonged starvation. However with regard to lipid content we also found a lowered proportion in the un-treated control group. Variable glycogen contents were detected (Fig. 4E). No decline in glycogen content was observed in starved flies, while in dehydrated flies there was a tendency towards lower glycogen content compared to in the control group.

## Discussion

As predicted by Rezende et al. [17] and Santos et al. [18] we found that thermal assays of heat tolerance that involved gradual heating (ramping assays) in a dry environment caused significant reductions in water content. This clearly demonstrates that ramping entails a dehydration event such that without water or food in the sealed test tube the water content of flies was decreased from around 0.8 to 0.7 mg H_2_O fly^−1^ after ramping (Fig. 3B). This water loss corresponded to 18% of the fly's initial water store. Importantly, we did not find empirical evidence for a negative effect of dehydration on thermal tolerance and this result was found regardless of the ramping rate employed (0.1 or 0.06°C min^−1^). In contrast to the modeled prediction of Rezende et al. [17] we actually found the flies ramped up in temperature without water or food had a slightly increased CTmax compared to those placed in empty vials. This finding is consistent with the results of Terblanche et al. [15] which also challenged the model predictions in Rezende et al. [17].

Rezende et al. [17] proposed that CTmax is reduced by the concurrent water loss such that the “true” CTmax is reduced by 0.05°C per % of total water lost. In our experiment this would correspond to a 0.9°C reduction in CTmax of flies ramped in empty vials relative to those that retain their water status in the food and agar vials. Instead we found that CTmax is actually increased by 0.2–0.3°C for flies ramped in empty containers. This seemingly positive effect of ramping in dry air is most likely a secondary effect of the evaporative cooling that must have occurred as a consequence of the water loss [20]. Based on these findings we conclude that this level of dehydration does not reduce CTmax during ramping.

We also addressed the predictions from the Rezende et al. [17] and Santos et al. [18] papers by another independent approach involving a short heat exposure (Fig. 4). Before we performed these test we exposed some experimental groups to a pretreatment consisting of either dehydration or starvation to test the predictions of the Rezende et al. [17] and Santos et al. [18] model. According to the results from their model dehydrated and/or starved flies should perform worse in these tests. However, we found no evidence to support this, suggesting that that the model or its assumptions are flawed. Our pretreatments did induce water loss and usage of glycogen/lipid reserves, but there was no difference in heat tolerance between the experimental groups. Clearly, within the range of dehydration and starvation stress induced in the present study there was no confounding effects on heat tolerance when assessed using acute short exposures to high temperature.

Overall our empirical findings do not agree with the conclusions of Rezende et al. [17] and Santos et al. [18] and we propose that this is related to the parameters used in their modeled predictions. In insects water is lost by respiratory, cuticular or excretion pathways [20], [21] where respiratory and cuticular water loss is driven by the partial pressure difference between the animal and its surroundings. In their models Rezende et al. [17] use data on water loss from Da Lage et al. [22] where flies are dehydration in completely dry air (silica gel) but as pointed out by Terblanche et al. [15] the “normal” experimental situation involves a much less stressful dehydration event since flies are introduced into experimental vials with a “normal” room humidity of 30–70% RH (See Fig. 2). Heating will, all else being equal, obviously entail an increasing partial pressure deficit of water as the drying power of the air increases with temperature [20]. However, in a closed system water loss from the insect will increase the surrounding partial pressure of water thereby dampening the otherwise increasing driving force for water loss at increasing temperatures. Because of these countering events it can be hypothesized that the driving force of water loss will increase, decrease or be constant as a temperature ramping event takes place. The outcome will ultimately depend on the size of the vial and on the total water conductance of the insect. If the thermal test involves exposure to a constant high temperature, then the driving force for water loss will actually decrease as time passes (and water is lost) during the test. In our system (see Fig. 2) the maximal amount of water that can be lost by evaporative means in our 5 ml experimental container is ∼0.25 mg if the maximal temperature reached is 40°C. This is obviously a worst-case scenario and as seen in our experiments water loss is actually lower. Furthermore some of this water will come from the metabolic water released during respiration, particularly if glycogen stores are used [23].

If dehydration is sufficiently severe then it will likely have a negative effect on animal performance in general, and on thermal tolerance specifically. As discussed in Terblanche et al. [15] such problems of dehydration are likely to increase as body-size decreases (see also [20]). Dehydration in a ramping test may therefore become a problem for smaller species or when using slower rates of temperature change. However, for *D. melanogaster*, the focal species of the Rezende et al. [17] and Santos et al. [18] papers, this is unlikely to be a problem. A recent study [14] investigated both acute (i.e. the “true” CTmax) and ramping CTmax of 10 *Drosophila* species. If dehydration during the test is a problem for this size class of insects then we would predict that the confounding effects of dehydration were larger in small flies (i.e. these flies would have a larger reduction in ramping CTmax *vs.* acute CTmax). However, this was not the case as the data actually show a non-significant trend in the opposite direction.

Rezende et al. [17] and Santos et al. [18] also suggest that depletion of energy stores can confound heat tolerance estimates during the course of a ramping test or a long term static test. However, in contrast to the predictions from these papers we found little evidence for depletion of energy reserves during ramping experiments. Thus, dry mass, lipid or glycogen reserves did not differ between treatment groups (Fig. 3C–E) nor were levels lower than in un-treated flies. Using a set of assumptions Rezende et al. [17] estimate an aerobic metabolism of up to ∼44 µL O_2_ for a 1 mg fly tested under conditions much similar to those used in the present study. This overall metabolism would correspond to the catabolism of 52 or 22 µg of carbohydrate or lipid, respectively [24] which should be seen as a detectable reduction in dry- glycogen- or lipid mass. Again we did not find support for this prediction. Thus, dry mass, glycogen and lipid content were not affected by the absence/presence of food, nor were these values lower than in un-treated control flies. In our experiment involving pre-exposures to starvation or dehydration we found reductions in dry, lipid and particularly glycogen content, but as discussed above neither of these changes were associated with a decrease in heat tolerance when tested using a short duration test. In conclusion there is no empirical evidence to suggest that starvation is a confounding factor in assessment of CTmax in *D. melanogaster* in ramping assays when employing rates of temperature changes ≥0.06°C min^−1^.

We propose that the discrepancy between the theoretical prediction and the empirical observation resides largely in the model assumptions in Rezende et al. [17] and Santos et al. [18]. For their calculations of metabolism during a ramping test Rezende et al. [17] used an estimate of metabolic rate of 4.2 ul O_2_ mg^−1^ h^−1^ at 18°C from Berrigan & Partridge [25]. This estimate of metabolic rate is in the high end of the literature values for *D. melanogaster*. After controlling for temperature using a Q_10_ of 2 we found that Djawdan et al. [26], Marron et al. [23], Gibbs et al. [21] and Overgaard et al. [27] all found estimates of metabolic rate that were 1.5–3 times lower than the estimate used by Rezende et al.[17]. The potential confounding effects of starvation are obviously very sensitive to the assumptions of the model and it is likely that a conclusion closer to the present empirical observations would have been reached using a different (and more realistic) set of assumptions. Along the same lines Santos et al. [18] used a Q_10_ estimate of 3.5. This estimate is very different from both the Q_10_ estimates of Berrigan & Partridge [25] and the Q_10_ of 2.1 found in *Drosophila* in the range between 16 and 36°C in our lab (Johannes Overgaard, unpublished observations). These estimates correspond to what is generally found for insects (Q_10_ of ∼2) (reviewed by [28]).

In addition to the proposed confounding factors of starvation and dehydration, Rezende et al. [17] argue that the ramping assay are not mimicking natural conditions precisely. Obviously we agree on this point but argue that this assay constitutes a step in the right direction for measuring ecologically relevant thermal tolerance in the laboratory. For some species and questions this might be an important consideration, whereas for other species and questions the method used might not be important. Arthropods undoubtedly experience major shifts in temperature on a daily and seasonal basis [15]. The gradual temperature changes that arthropods are exposed to in nature are in sharp contrast to laboratory test conditions in static thermal assays where animals are exposed to abrupt temperature changes, e.g. transferred acutely from rearing temperatures around 20–25°C to subzero temperatures or temperatures close to their upper thermal limit [29]. We argue that ramping assays are more ecologically relevant than static assays as they will better portray the thermal tolerance of animals in the field, e.g. better reflecting diurnal variation (see [4], [15], for a discussion of this). Several recent studies have shown that results obtained from traditional assays of thermal resistance do not reflect ecological relevance. Release-recapture studies have revealed that performance under natural conditions can deviate strongly from results obtained based on traditional laboratory assays [30], [31] (but see [32]). These findings emphasize the importance of developing more ecological relevant thermal laboratory assays and ramping assays constitute one such method that is useful for some animals, including drosophilids.

In accordance with earlier studies we found that slower ramping rates resulted in lower estimates of CTmax [4], [11], [15], [16]. We propose a simple alternative explanation for these findings. Imagine that over a given threshold, flies start to accumulate heat damage. It is for example well established that heat shock proteins are induced over a given temperature [33]. The further the animal is above this threshold the faster the accumulation of damage and similarly the longer duration over the threshold the more damage accumulates. CTmax will then represent a measure of when a certain amount of damage is accumulated. This simple model could be expanded with inductions of protective mechanisms (hardening) and thresholds for damage may also be changed by thermal acclimation. This simple model can also explain reduced CTmax as a function of duration and ramping rate and it can do so in a manner that is independent of putative confounding effects of dehydration and starvation.

Although we fully appreciate the potential problems of dehydration and starvation highlighted by Rezende et al. [17] and Santos et al. [18] we find no support for the conclusions in these papers when testing *D. melanogaster* using ramping rates of 0.06 or 0.1°C min^−1^. We encourage researchers to continue to consider the general experimental conditions experienced in laboratory assays of thermal resistance. As exemplified in Fig. 2 this is done most easily by appreciating the starting conditions for an experiment and then giving some thought to how these conditions may change over the course of the thermal tolerance test. Even better, researchers could quantify experimentally the putative impact of e.g. starvation, dehydration, hypoxia and hypercapnia to investigate if these factors constitute a problem in relation to the interpretation of results obtained from thermal tolerance assays.
